# Case report: Congenital absence of the dens

**DOI:** 10.4103/0971-3026.63050

**Published:** 2010-05

**Authors:** Manish Bajaj, Hemant Jangid, AK Vats, ML Meena

**Affiliations:** Department of Radiodiagnosis, R.N.T. Medical College, Udaipur, Rajasthan - 313 001, India; 1 MR Centre, GBH American Hospital, Udaipur, Rajasthan - 313 001, India; 2 Neurology Centre, 21-C A Madhuban, Udaipur, Rajasthan - 313 001, India

**Keywords:** Absent, congenital, dens

## Abstract

A 36-year-old man presented with headache and right upper and lower limb weakness for 10 days. MRI revealed absence of the odontoid process of the C2 vertebral body, with resultant atlantoaxial dislocation along with myelomalacic changes involving the cervicomedullary junction.

## Introduction

Developmental anomalies of the odontoid are uncommon. The clinical importance lies in their potential to cause serious neurological complications due to atlantoaxial instability.

We report a case of congenital absence of the dens, with resultant atlantoaxial dislocation and partial fusion of the C2 and C3 vertebrae.

## Case Report

A 36-year-old man presented with a history of minor trauma to the head following a sudden jerk while traveling in a bus. He complained of headache and right upper and lower limb weakness. On examination, power in the right upper and lower limbs was 4/5. The right plantar reflex was extensor. No sensory deficit or bladder/bowel involvement was seen.

**Figure 1:**
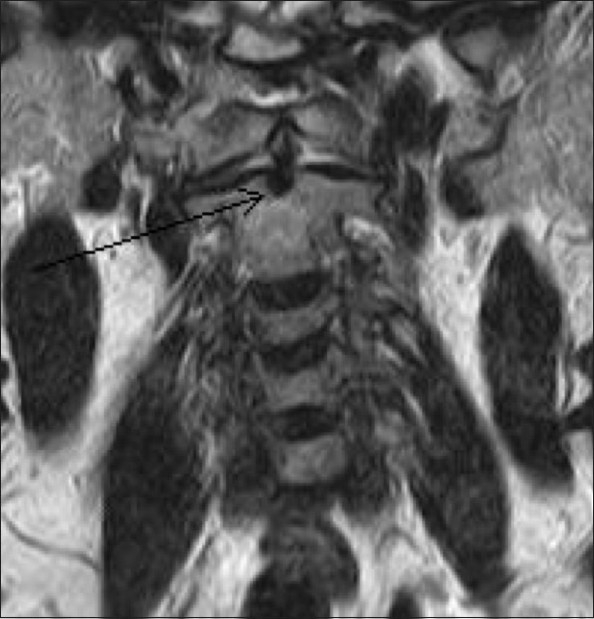
Coronal T2W MRI image shows a well-defined depression (arrow) on the superior aspect of the body of C2 with non-visualization of the dens

**Figure 2:**
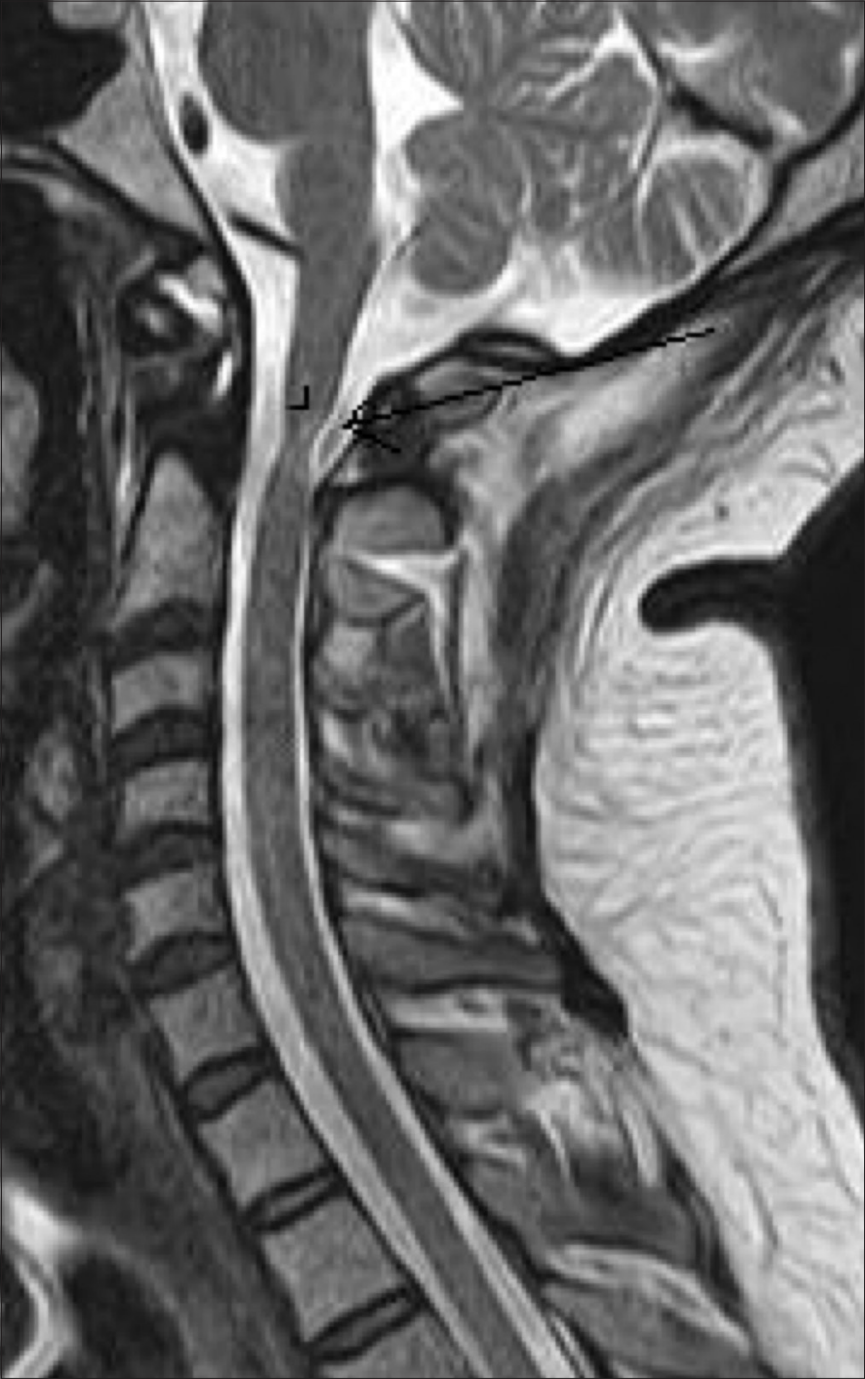
Sagittal T2W MRI image shows reduced caliber of the cervical spinal cord (arrow) with a hyperintense intramedullary signal (arrowhead) at the C1-C2 level

**Figure 3:**
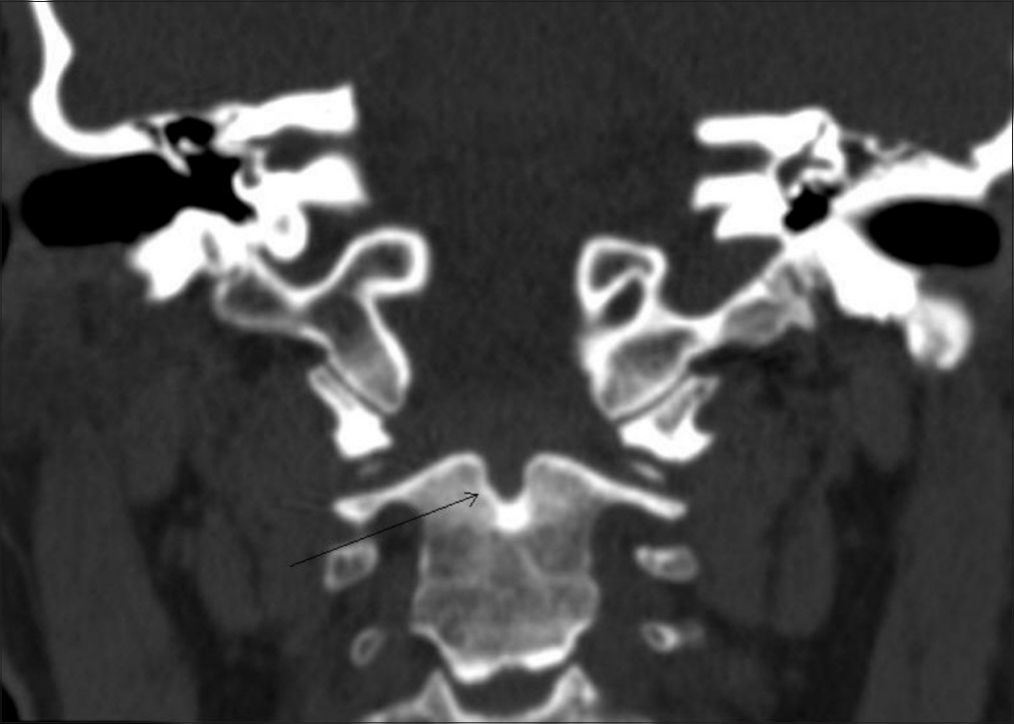
Coronal CT scan reconstruction shows a well-corticated depression (arrow) on the superior aspect of the body of C2, with non-visualization of the dens

**Figure 4:**
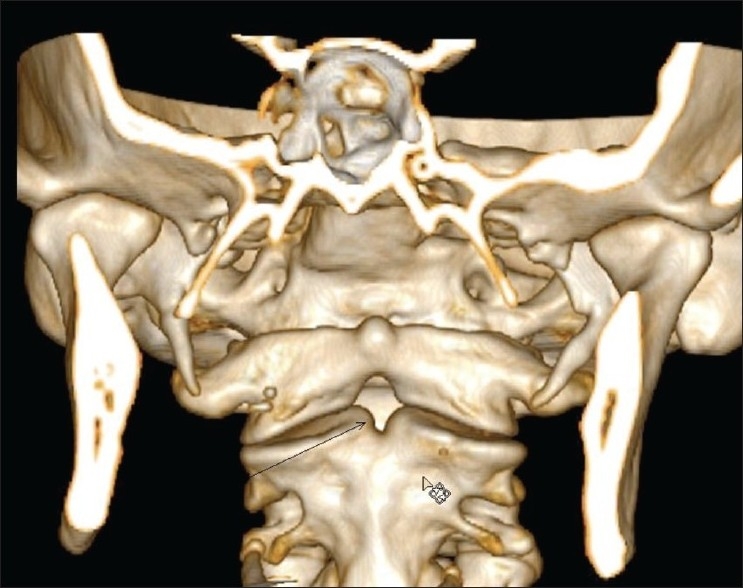
Three-dimensional volume-rendered post-processed CT scan shows absence of the odontoid process (arrow)

MRI of the brain and the cervical spine was performed, which revealed absence of the odontoid process of the C2 vertebra [Figure 1], with resultant atlantoaxial dislocation [Figure 2]. There was reduced caliber of the spinal cord at the C1-C2 level, with T2 hyperintense signals, suggestive of myelomalacic changes [Figure 2]. Subsequent non-contrast CT scan of the cervical spine with sagittal and coronal reconstructions confirmed the absence of the dens; there was also a well-corticated depression involving the superior border of the body of C2 [Figure 3]. Also noted was fusion of the body and posterior elements of the C2 and C3 vertebrae and subtle anterior migration of the axis vertebra at the atlanto-occipital junction. Three-dimensional volume-rendered post-processed CT scan [Figure 4] depicted the anomaly elegantly.

## Discussion

Congenital anomalies involving the craniovertebral junction are clinically important because of their potential for producing serious neurological deficits. The clinical features are chiefly because of the associated atlantoaxial dislocation and include persistent neck pain, headache, and transient/permanent paresis.[1] Often, patients presenting for the evaluation of trauma are found to have such anomalies, incidentally.

Congenital anomalies involving the odontoid process are rare. They can be classified into os odontoideum, ossiculum terminale, aplasia-hypoplasia,[2] and duplication of the dens.[3] Of these, aplasia of dens is very rare, with only a few cases reported in the literature.[4]

**Figure 5:**
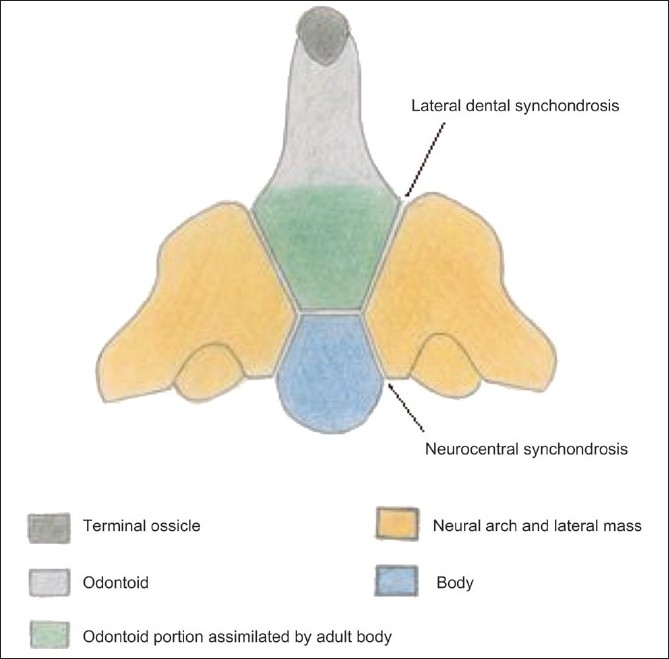
Line diagram of the C2 vertebra with its ossification centers

The development of the axis is complex [Figure 5]. Four ossification centers are present at birth: one for each neural arch, one for the body, and one for the odontoid. In utero, the dens has two ossification centers in the midline, which fuse by the 7th month of intrauterine life. A secondary ossification center appears at the apex (the terminal ossicle) at 3–6 years of age and fuses with the rest of the dens by 12 years of age. The body of C2 normally fuses with the dens by 3–6 years, with obliteration of the subdental synchondrosis. Posteriorly, the neural arches fuse by 2–3 years of age, and fusion with the odontoid takes place between 3 and 6 years of age, with obliteration of the neurocentral and lateral dental synchondrosis [Figure 5].[5–10]

In our case, the dens itself, as well as the part of the dens assimilated by the body of C2, was absent [Figure 5], the latter being evident by the corticated depression in the upper part of the C2 body in the midline.

Anomalies of the odontoid can usually be diagnosed on a standard series of radiographs, plus flexion and extension lateral films. With the increasing importance of CT scan and MRI in the study of the craniovertebral junction, a detailed evaluation of such congenital anomalies is now possible, as demonstrated in our case.

The first case of odontoid hypoplasia was reported by Roberts in 1933.[11] Since then, hypoplasia has been reported a few times but aplasia is considered very rare.[12]
